# Prototype of a Textronic Sensor Created with a Physical Vacuum Deposition Process for *Staphylococcus aureus* Detection

**DOI:** 10.3390/s21010183

**Published:** 2020-12-29

**Authors:** Ewa Korzeniewska, Artur Szczęsny, Piotr Lipiński, Tomasz Dróżdż, Paweł Kiełbasa, Anna Miernik

**Affiliations:** 1 Faculty of Electrical, Electronic, Computer and Control Engineering, Lodz University of Technology, Stefanowskiego 18/22, 90-924 Łódź, Poland; artur.szczesny@p.lodz.pl; 2 Faculty of Technical Physics, Information Technology and Applied Mathematics, Lodz University of Technology, ul. Wólczańska 215, 90-924 Łódź, Poland; piotr.lipinski@p.lodz.pl; 3 Faculty of Production and Power Engineering, University of Agriculture in Krakow, Balicka 116 B, 30-149 Kraków, Poland; tomasz.drozdz@office.urk.edu.pl (T.D.); pawel.kielbasa@urk.edu.pl (P.K.); miernikanna07@gmail.com (A.M.)

**Keywords:** physical vacuum deposition, biosensor, wearable electronics, *Staphylococcus aureus*, textile composite, bacteria detection

## Abstract

*Staphylococcus aureus* is a bacterium which people have been in contact with for thousands of years. Its presence often leads to severe disorders of the respiratory and circulatory systems. The authors of this article present a prototype of a textronic sensor enabling the detection of this bacterium. This sensor was created using a process of physical vacuum deposition on a flexible textile substrate which can be implemented on clothing. With increasing numbers of bacterial colonies, changes in the sensor’s electrical parameters were observed. The sensor’s resistance reduced by 50% and the capacitance more than doubled within the first two days of starting bacterial cultures. Extensive changes in electrical parameters were observed at 100 Hz and 120 Hz of the measurement signal.

## 1. Introduction

Human skin is the largest organ of the body, constituting a barrier against microorganisms, including those of a pathogenic nature [1]. Many microorganisms inhabit human skin, with the most numerous being bacteria and fungi. These microorganisms can be divided into two groups: permanent and transient (periodically appearing) microflora. The presence of auto- and allochthonous flora on the skin depends on many environmental and individual factors [2]. Higher temperature and humidity in certain areas (e.g., the groin or armpit) favor the development of microorganisms, mainly Gram-negative bacteria or *Staphylococcus aureus* [1,3,4,5,6]. Using genetic engineering, it has been established that the solid microflora of the skin include coagulase-negative staphylococci and bacteria of the genera *Corynebacterium, Propionibacterium, Brevibacterium*, or *Micrococcus*. In contrast, transient colonizers (mainly in pathological conditions) include *Staphylococcus aureus, Staphylococcus pyogenes*, and *Pseudomonas aeruginosa* [1,5,7,8,9].

Skin infections are most often caused by staphylococci and streptococci [10]. *Staphylococcus aureus* is the only coagulase-positive staphylococcus commonly found on the skin and mucous membranes of humans. Usually, it does not appear on a healthy person’s skin, but it inhabits the mucous membranes of the nose and throat. According to multiple studies in healthy human populations, between 18% and 40% are carriers of *Staphylococcus aureus*. It is believed that about 20% of the population are persistent carriers, with some being asymptomatic [6,11,12,13].

Conventional *Staphylococcal aureus* detection methods are laborious and require laboratory work. Among them there is a molecular biology analysis based on the polymerase chain reaction, which is an indirect method of microbial detection. The available chromatographic methods require trained personnel and tedious pre-treatment of the sample. In line with global trends, the aim should be to develop sensors based on new technologies, such as immunosensors, in combination with optical, electrical, and mass detection techniques. A detailed description of *Staphylococcal aureus* detection procedures is provided by Wu et al. [14].

Practical determination of a microorganism’s presence requires analyses, for which the main limitations are the time and sensitivity of the method. Biosensors are a promising alternative tool for the rapid detection of microorganisms, and because of their potential, they are used in various spheres of human life [15].

The authors of this paper present a textronic sensor prototype that can be integrated into human clothing. Such sensors can be part of a wearable electronic system.

**Figure 1:**
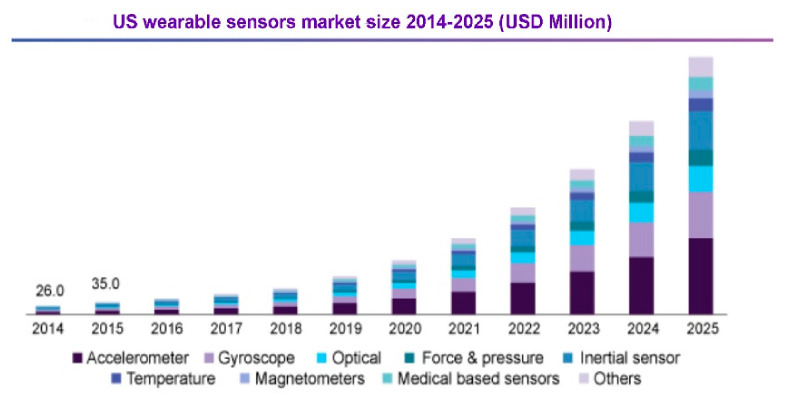
The expected increase in the value of the sensor market [16].

Wearable electronics were originally developed the second decade of the twentieth century. As a precursor, in his laboratory at MIT (Massachusetts Institute of Technology) Steve Mann worked on reducing electronic devices to a portable size. Nowadays, with the miniaturization of electronic components at an advanced level, scientists are combining miniaturized electronic systems with human clothing. Therefore, a new scientific field was created to combine electronics, material engineering, metrology, and computer science with textiles, with the result being textronics. This is also an area where sensors will thrive. According to data from 2018, the wearable sensors market in 2016 reached around US$150 million, and it is expected to increase (Figure 1).

The combination of textile substrates with electronic systems elements is possible using one of many methods including embroidery, knitting, weaving, ink-jet printing, dip-coating, electroless plating, magnetron sputtering, chemical vapor deposition, or physical vacuum deposition [17,18,19,20,21,22,23]. Depending on the used method, it is possible to produce electrically conductive structures at various stages when creating the final textile product. The use of individual methods also depends on the physicochemical properties of the textile substrate.

There are known sensors based on flexible substrates made of textiles. However, most often they use electrically conductive fibers embedded in the fabric structure using the process of weaving, knitting, or embroidery [24]. Flexible substrates facilitate diagnostics, improving non-invasive health monitoring. A review of the available solutions of biosensors produced on flexible polymer substrates is described in [25]. Mathew et al. described the possibility of using flexible, short-term disposable sensors on other polymer substrates to monitor health in real time, using physiological fluids such as saliva, sweat, and tear fluid (e.g., contact lenses for glucose monitoring) [26].

The authors then describe the results of their work in relation to the sensor’s production for bacteria detection on a textile substrate. The sensor structure was developed based on interdigitated electrodes (IDEs) on a flexible substrate that can be an integral part of any textile product [27].

IDEs are usually produced on rigid substrates such as silicon or glass [28,29,30]. Interdigitated electrodes are made in the form of two individual addressable comb-like interdigitated structures. IDEs form part of a group of transducers widely used in metrology where impedance and capacity changes are essential, e.g., in humidity and gas sensors, and in biosensors for tumor cell and cardiac troponin T biomarker detection or in biomedical applications [31,32,33,34,35,36,37,38,39,40]. In the literature, there are also reports on the usage of IDEs in sterility tests in the food industry. One sensor was used to assess sterilization processes using H_2_O_2_ (hydrogen peroxide) vapor [29]. The authors of the work suggested using a sensor built based on IDEs for the detection of bacteria, specifically *Staphylococcus aureus*, which can be present on the skin. The sensor should be a part of specialized dressings for difficult-to-heal wounds [41], which are most often changed every 3 days. The location of the sensor in such a dressing will allow not only for tracking potential microbiological hazards, but also for monitoring the treatment process and generating an alarm in abnormal conditions. In the future the authors also will conduct a study using IDEs sensors for a broader range of temperatures, as in [42,43].

## 2. Materials and Methods

### 2.1. Sensor Preparation

The interdigitated electrodes were prepared and used as a sensor for bacteria detection. They were manufactured in an environmentally friendly, physical vacuum deposition process (PVD) using silver with high purity (99.99% guaranteed by the Metals Mint Ltd., Poland) as the deposited metal. The authors investigated sensors with silver layers because this metal is known for its antibacterial properties [44] and as a material which is stable in air with a wide temperature range [45]. The electrodes were created on a textile composite substrate known on the market as Cordura. It is the trade name for a composite made from nylon threads covered with a polyurethane layer which produces an electroconductive continuous layer. The surface mass of the used substrate was 195 g/m^2^.

**Figure 2:**
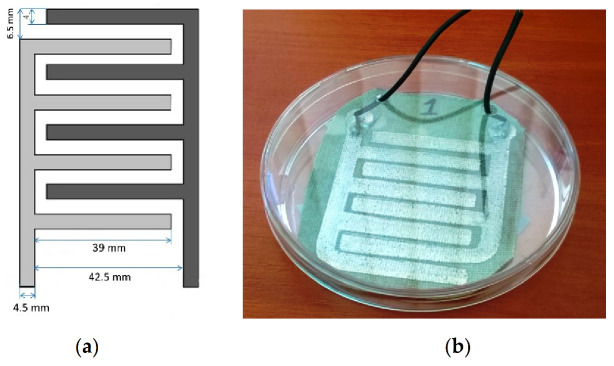
(**a**) Geometry of the produced interdigitated electrodes (IDEs); (**b**) photo of the produced structure.

The pre-vacuum value in the PVD process was 5·× 10^−5^ mbar (0.005 Pa). The application time of the metal from the tungsten boat source was 5 min. The substrate was cleaned with organic solvents and then conditioned at 23 °C and 50% humidity for 24 h. To obtain the assumed shape, masks with the geometry shown in Figure 2 were prepared. The usage of an obscuring mask to produce the desired structures is associated with the phenomenon of shading at the area between the mask and the substrate. For that reason, to minimize this undesirable effect, the authors used a 1-mm-thick mask made of stainless steel created using a laser cutting process [46].

The authors of the study placed measuring electrodes using electroconductive AMEPOX adhesive glue on the bottom of the electrodes (Figure 2b).

### 2.2. Bio Material

In known evaluation research methods, detection of *Staphylococcus aureus* is recommended to test infertility (the fertility of substrates used and the suitability of the method), the microbiological cleanness of non-vital products (quantitative microbiological qualities), the effectiveness of disinfectants, and protection against microbes (maintenance test) (Table 1).

For that reason, the authors of the article chose the *Staphylococcus aureus* bacterium to test the suitability of textronic sensors for bacteria detection. The choice of *Staphylococcus aureus* is additionally justified due to the nature of the selected substrate and the possibility of using it as an integral part of clothing or other textiles.

The strain *Staphylococcus aureus* ATCC 25923 was used as an example of the microorganism of transient skin microflora. The used reference strain was obtained directly from the American Type Culture Collection (ATCC, Manassas, Virginia). It belongs to a group of microorganisms that cause exogenous nosocomial infections. Before testing, the strain was stored in a specialized microbank in a frozen state (−70 °C) in the form of a lyophilizate. Strains which are stored in such conditions for many years do not change their properties during storage.

At the initial stage of research, the substrates were autoclaved at a temperature of 121 °C, with a pressure of 1 bar for 15 min. Before testing, the strains were revived by spreading on a Petri dish with solid trypticase soy agar (TSA) medium (Trypticasein Soy LAB-AGAR, BIOMAXIMA, POLAND), with the composition listed in Table 2. It is a non-selective, tryptose-soybean medium for the general cultivation of microorganisms with different growth requirements from clinical samples, pharmaceutical preparations, cosmetics, etc. Cultivation was carried out under aerobic conditions for 24 h at 37 °C.

### 2.3. Measurement Method

**Figure 3:**
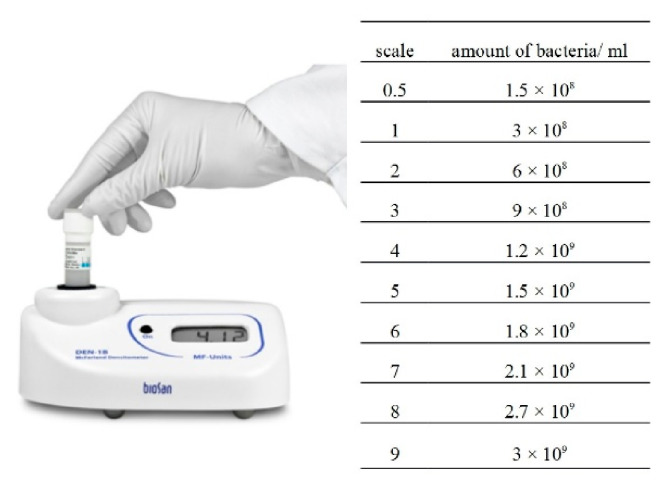
McFarland scale.

One-hundred-and-fifty milliliters of sterile liquid nutrient medium Nutrient Broth (BIOCOPR, Warsaw, Poland) were poured to the sterile conical flasks (300 mL). The composition of the medium is presented in Table 3. It is a liquid for general use, mainly for the propagation of microorganisms with low growth requirements. Then, from the obtained colonies, a test culture was prepared by preparing microbial suspensions with an optical density of 0.5 on the McFarland scale (Figure 3).

The optical density was measured with a DEN-1B densitometer. Then, in separate, sterile, glass Petri dishes (d = 200 mm) the tested sensors were placed and flooded with previously prepared microbial suspensions.

### 2.4. Electrical Measurement

Measurements of electrical capacitance and resistance as a real component of impedance using the CEM DT-9935 impedance meter were taken in the case of clean sensors, sensors placed in clean medium, and those placed in the microbial suspensions with an initial optical density of 0.5 on the McFarland scale. The meter uses the ES51919/ES51920 chipset, which includes an analogue front-end integrated circuit with a resistor network to control various ranges. The circuit also provides a variable frequency generator for complex impedance measurement. It is equipped with a 4.5 digital A/C converter. The circuit has the ability to measure AC impedance and DC resistance. Thanks to this, it is possible to automatically or manually measure inductance and capacitance together with parasitic resistances as well as the dissipation factor (D), Q factor (Q), and phase angle of impedance. It is also possible to determine the equivalent resistance of the equivalent circuit of a series or parallel capacitor and coil. The test frequencies of 100 Hz/120 Hz/1 kHz/10 kHz/100 kHz can be selected depending on the tested structure type. Due to the capacitive nature of the circuit, the authors used the measurement at alternating current to obtain the real part’s value (resultant resistance) and the reactance (imaginary part) of the thin-film structure. The necessity to carry out R and C measurements at alternating current also results from the skin effect for the thin-film structure and its influence on the real part of impedance as a function of frequency. Due to the low values of the measured capacitance, capacitance measurements were taken in a parallel system following the meter manufacturer’s recommendations. The measurements were taken at the beginning of the tests and 21, 28, 45, 52, 69, 77 and 90 h after establishing the culture. Cultures were grown on a shaker (50 rpm) at room temperature.

## 3. Results and Discussion

Silver is a metal with a broad spectrum of anti-microbial activity, and at the same time, it exhibits low toxicity towards mammalian cells. Because of the interaction of silver with bacteria, creating sensors based on that metal is justifiable. The sheet resistance value of the produced electroconductive structures was 1.2 Ω/sq.

**Figure 4:**
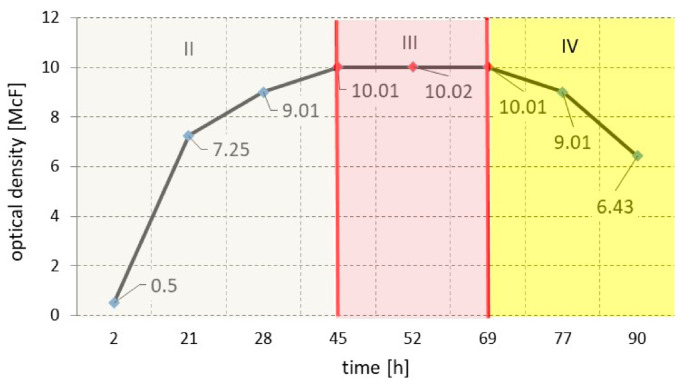
The curve of bacterial growth of *Staphylococcus aureus* ATCC 25923.

During the tests, in addition to electrical parameters, bacterial growth was also monitored using a standard measurement procedure—the McFarland scale. The plot of the number of bacteria as a function of time is presented in Figure 4. The measurements made indicated the development of the bacteria. Changes in dielectric properties between flat electrodes will cause a change in the capacity as well in the resistance of the entire IDE structure.

The growth process of the tested strain of *Staphylococcus aureus* ATCC 25923 is represented by a growth curve. For certain culture conditions, the bacterial growth curve is characteristic for the species. Figure 4 shows three of the four theoretical phases of microorganism development. Phase II is the exponential growth phase (logarithmic phase). The bacteria divide very quickly, and during this phase, the cell suspension should be taken as the initial sample for subsequent dilutions. Phase III, i.e., the stationary phase, is characterized by a constant number of cells in culture due to the decreasing number of nutrients and the increasing amounts of metabolite in the medium. Phase IV is the dying out phase (final phase), where the quantities of metabolic products in the medium becomes toxic, and the lack of nutrients leads to the death of the culture.

**Figure 5:**
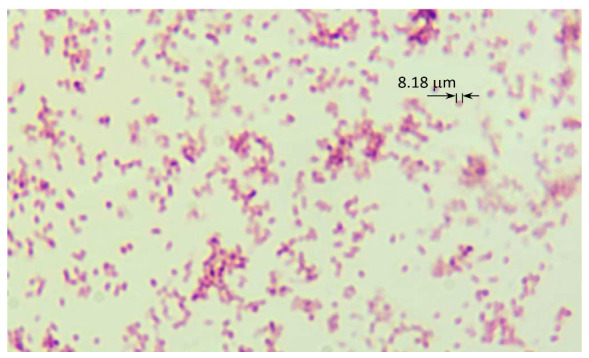
The microscopic image of suspension with *Staphylococcus aureus*—optical density 6.25 McF.

In the third phase of bacterial growth, optical density stabilized at the level of 10 McF, which was recorded between 45 and 69 h of bacterial growth. Figure 5 shows an image that was taken via an optical microscope when the optical density was 6.25 McF.

**Figure 6:**
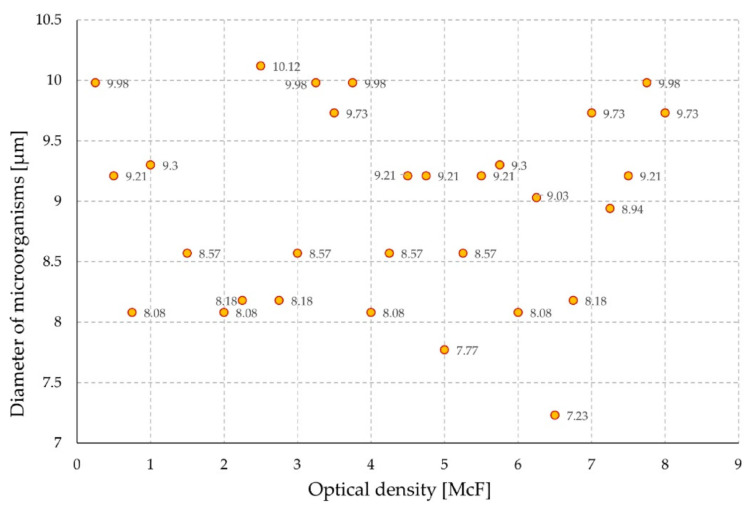
The diameter of microorganisms in relation to the optical density of the suspension.

The geometrical parameters of individual microorganisms were examined at different levels of optical density of the solution to exclude its influence on the results of sensor’s electrical measurements. The tests were performed on a suspension without placed sensors. There was no effect of optical density on the cell diameter (Figure 6).

**Figure 7:**
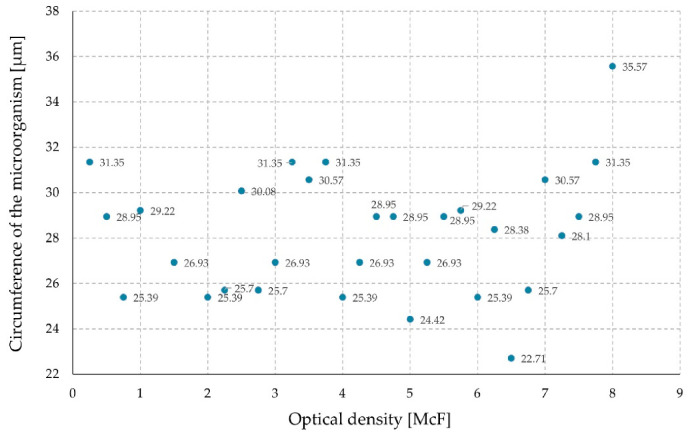
Circumference of the microorganism in relation to the optical density of the suspension.

Additionally, the circumference of the microorganism was measured (Figure 7). No quantitative relationships were found.

**Figure 8:**
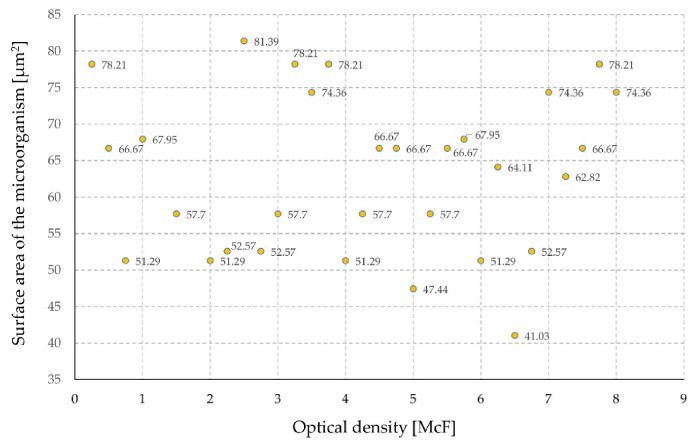
The surface area of the microorganism in relation to the optical density of the suspension.

The surface area of the microorganism was also determined (Figure 8). As in the above-mentioned cases, the influence of the surface of the microorganism on the optical density and the measurement result was excluded in the case of sensor tests.

Because there was no difference in the geometric dimensions of bacteria (Figure 6, Figure 7 and Figure 8), the time of the experiment was determined only by the optical density of the suspension.

**Figure 9:**
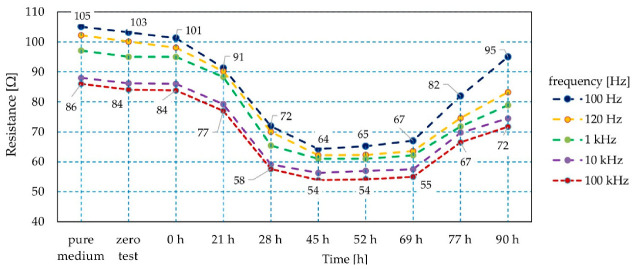
The resistance characteristics of the textile sensor during bacterial colony development.

The quantitative identification of bacteria is a critical issue in terms of human safety. Therefore, it should correlate with textile sensor output. Figure 4 shows the sensor resistance (the real component of impedance) characteristics as a function of bacterial growth time (see Figure 9). An evident relationship between the number of bacteria and the sensor characteristics was found. The resistance value decreased (by 50%) with the increased optical density of the preparation, indicating that the number of bacteria was higher. Therefore, based on resistance measurement in the analyzed combination of experiments, it is possible to estimate the dynamics of bacterial growth and to identify the development phases. The presented results show the average values of measurement with 20 sensor samples.

During the research, the relationship between the resistance value and frequency was observed, i.e., the higher the frequency, the lower the resistance in the entire measuring range. The listed differences are relatively constant, but the smallest ones were in the third phase of bacterial growth, i.e., the stabilization phase. The relative difference between resistance values at extreme frequencies was about 16%.

**Figure 10:**
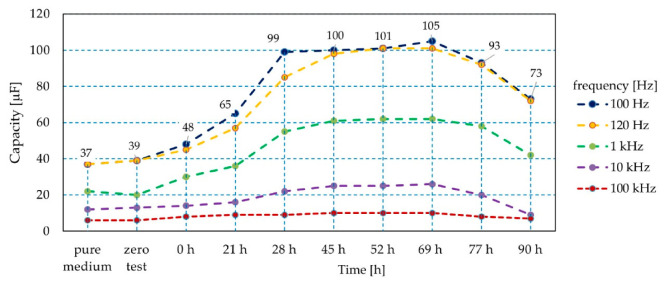
Characteristics of the textile sensor capacitance during bacterial colony development.

A slightly different observation concerns the measurement of the textile sensor capacitance in the environment of *Staphylococcus aureus*, where a higher concentration of bacteria generated a higher value of the textile sensor capacitance (Figure 10). The sensor electrical capacitance was measured in equal intervals of time. In Figure 5, those points were connected with a broken line, which allows straightforward interpretation of the relationship between the sensor capacitance and the number of bacteria. As in the case of the sensor impedance value, it is possible to identify the dynamics of bacterial growth detailing the colony growth phases, which can be used directly in practice. Of note was the differentiation of the measured sensor capacity depending on the number of bacteria, where the minimum value was several dozen μF and the maximum number of bacteria was three times higher. This applied to all measured frequency ranges except for the frequency of 100 kHz.

It was observed that in the case of an effective method of quantitative identification of staphylococci based on the textile sensor capacitance, frequencies not exceeding 1 kHz should be used when the diversity concerning changes in the number of bacteria is clear and easily identifiable.

**Figure 11:**
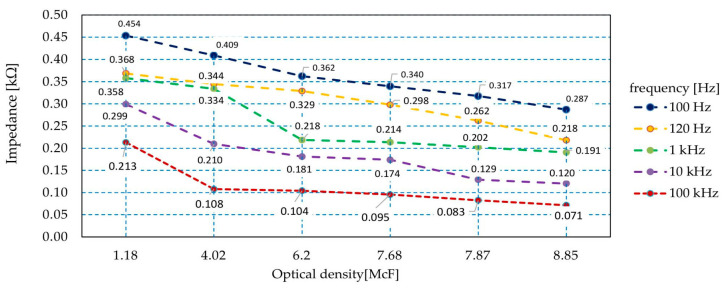
The dependence of impedance of the sensor with regard to the number of bacteria in the suspension.

**Figure 12:**
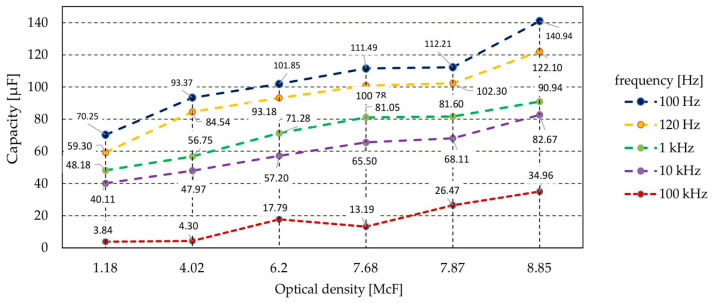
The dependence of the sensor capacity with regard to the number of bacteria in the suspension.

The sensor’s capacity and impedance were measured with regard to microorganism quantity in the initial phase of growth. The measurement results are presented in Figure 11. The increase in the number of bacterial cells in the suspension caused a reduction in the impedance value of the produced sensor. Impedance changes of 40% or more were observed throughout the measurement range. With the measuring signal at the frequency of 100 Hz/120 Hz, the impedance values were twice as high as with higher frequency values (Figure 11). Changes in values were also observed during the measurement of the capacity of sensor structures. In this case, with the growth of bacteria, the capacity with regard to parasitic IDE structures increased. For the lower frequencies of the measurement signal, a 100% increase in capacity was observed. For the higher frequencies used in the experiment, this increase was by as much as nine-fold (Figure 12).

In order to determine the sensitivity of the proposed sensory element, the impedance was also measured in a distilled water solution and in an environment of bacteria harmless for human health such as *Lactococcus lactis* LI-23, *Bifidobacterium longum* BI-05, *Lactobacillus helveticus* SP27, *Lactobacillus plantarum* Lp-115, *Lactobacillus casei* Lc-11, *Lactobacillus rhamnosus* Lr-32, and *Bifidobacterium bifidum* Bb-02. These are present in Multilac Symbiotic (USP Zdrowie Ltd., Warsaw, Poland). The properties of the sensor were tested at an optical density of 4.94 McF.

**Figure 13:**
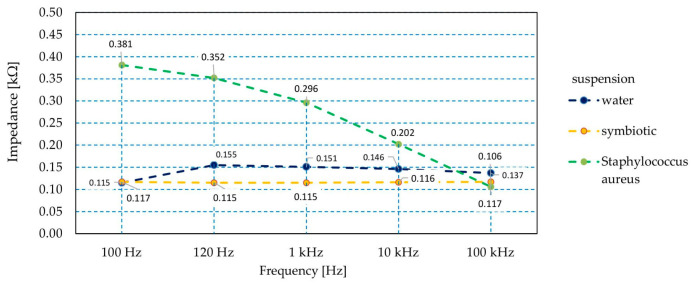
The dependence of the sensor impedance with regard to frequency in various types of suspensions.

Significant differences were observed between the impedance values for beneficial bacteria and water as well as for *Staphylococcus aureus*, reaching 300% at 100 Hz and over 200% at 120 Hz. The increase in the frequency of the measurement signal reduced the differences in the values of the measured impedance of the sensor immersed in the tested solutions (Figure 13). Thus, there was a difference in the impedance indications of the sensor immersed in demineralized water and a suspension of Multilac Symbiotic of 20% at frequencies higher than 100 Hz. There was a clear distinction between the sensor impedance readings when in contact with harmful and beneficial microorganisms.

## 4. Conclusions

Despite advances in medicine, *Staphylococcus aureus* is still a threat to humans. The use of silver as an antibacterial metal is common. In this article, the interaction between silver and *Staphylococcus aureus* was used to detect the presence and also measure the numbers of bacteria. The increase in the number of bacteria caused changes in the electrical parameters of the produced sensor. The most extensive changes in electrical parameters were observed at 100 Hz and 120 Hz. Changes in the resistance values and sensor capacitance were observed at as early as the first day of testing. This information can be useful for professionals in order to decide how to treat patients. The most extensive parameter changes occurred within the 28–69 h time window from initial bacterial infection. The desired changes in electrical parameters were observed in the initial phase of the multiplication of the *Staphylococcus*. This allows for quick detection at the point of contact of the measurement electrodes with the undesirable microorganism. Due to the nature of the substrate used to produce the sensor, it can be implemented in various types of textile products.

## Figures and Tables

**Table 1:** Standard strains recommended in microbiological methods.

Methodology	Recommended Reference Strains of Aerobic Bacteria
Infertility To test the fertility of substrates used and the suitability of the method	*Staphylococcus aureus* *Bacillus subtilis* *Pseudomonas aeruginosa*
To study the microbiological cleanness of non-vital products (quantitative microbiological qualities)	*Staphylococcus aureus* *Bacillus subtilis* *Pseudomonas aeruginosa*
To determine the effectiveness of protection against microbes (maintenance test)	*Staphylococcus aureus* *Pseudomonas aeruginosa*
To test the effectiveness of disinfectants	*Staphylococcus aureus* *Pseudomonas aeruginosa* *Enterococcus hirae* *Escherichia coli*

**Table 2:** The composition of TSA medium.

Ingredient	Value (g/L)
Pancreatin casein hydrolysate	15
Soy peptone	5
Sodium chloride	5
Agar	15

**Table 3:** The composition of Nutrient Broth Medium.

Ingredient	Value (g/L)
Peptone	5
Beef extract	3

## Data Availability

The data presented in this study are available on request from the corresponding author. The data are not publicly available due to privacy.
